# HLA II class alleles in juvenile idiopathic arthritis patients with temporomandibular joint involvement

**DOI:** 10.1186/1546-0096-12-S1-P24

**Published:** 2014-09-17

**Authors:** Zane Davidsone, Elena Eglite, Sarmite Dzelzite, Arina Lazareva, Ruta Santere, Dace Berzina, Valda Stanevicha

**Affiliations:** 1 Paediatric Department, Riga Stradins University, Riga, Latvia; 2 Laboratory of Clinical Immunology and Immunogenetics, Riga Stradins University, Riga, Latvia; 3 Radiology Department, Children University Hospital, Riga, Latvia; 4 Rheumatology Department, Children University Hospital, Riga, Latvia

## Introduction

Temporomandibular joint (TMJ) involvement is seen very often (17-87%) in children with juvenile idiopathic arthritis that can lead to compromised craniomandibular function, dentofacial aestethics and morphology. Contrast enhanced MRI is the golden standart for diagnosis of TMJ arthritis (Argyropoulou, 2009). Previous studies show that HLA II class alleles may have protective or risk importance in JIA subtypes (Hollenbach, 2010).

## Objectives

To identify HLA II class alleles of risk and protection in JIA patients with TMJ involvement.

## Methods

53 JIA patients were evaluated treated at Children University Hospital in whom MRI for TMJ from 2010.-2014 was performed. Patients were genotyped for HLA- DRB1; DQB1; DQA1- using RT-PCR with sequence-specific primers. Associations of DRB1; DQB1; DQA1 alleles in patients were examined individually using the χ^2^ test. P-value and odds ratio were calculated using EPI INFO 6.0 software with 95 % confidence intervals and Fisher correction for small numbers.

## Results

53 JIA patients with mean age of 14,67 ±1,15 years (range 1,15 – 17,9 yr); 39 (73,6%) girls and 14 (26,4%) boys. The mean duration of the disease from the time of diagnosis till performing TMJ MRI was 3,96 ±2,22 years (range 0,2 – 10,2 yr). JIA subtype were as follows: seronegative polyarthritis 29 (54,7%), seropositive polyarthritis 6 (11,3%), olygoarthritis extended 4 (7,5%), arthritis with enthesitis 9 (17%), undifferentiated 2 (4,75%) and 2 (4,75%) for systemic arthritis. 2 groups where separated after TMJ MRI: 1st with ≥2 signs of active inflammation or any structural damage; 2nd with no pathologic signs or with slight contrast enhancement. In the 1st group alleles DRB1 *16:01 (OR 0,90, p=0,0001), *13:01 (OR 6,34, p=0,01); DQB1 *02:01-02:02 (OR 3,3. p=0,001); DQA1 *02:01 (OR 6,39, p=0,041) where observed. In the 2nd group DRB1 *11:01(OR 0,17, p=0,0001); DQB1 *03:01 (OR 0,3, p=0,005), *05:01 (OR 0,4, p=0,026); DQA1 *05:01 (OR 0,22, p=0,001) were found more often.

## Conclusion

1) JIA patients with alleles DRB1 *16:01, *13:01; DQB1 *02:01-02:02 and DQA1 *02:01 may have higher risk for TMJ involvement with 2 or more signs of active joint inflammation or any structural damage. 2) DRB1*11:01; DQB1 *03:01, *05:01 and DQA1 *05:01 alleles are probably protective for TMJ involvement.

## Disclosure of interest

None declared.

